# Adolescent Symptomatic Medial Meniscal Ossicle Treatment and Probable Etiology

**DOI:** 10.7759/cureus.67411

**Published:** 2024-08-21

**Authors:** Kevin Adik, Melanie A Morscher, Andrew J Newsom, Kenneth Bono

**Affiliations:** 1 Department of Orthopedics, McLaren Flint Hospital, Flint, USA; 2 Department of Orthopedics, Akron Children's Hospital, Akron, USA; 3 College of Medicine, Northeast Ohio Medical University, Rootstown, USA

**Keywords:** medial meniscal ossicle, arthroscopy, partial medial meniscectomy, adolescent, case report, pain

## Abstract

Meniscal ossicles are rare, especially in children and adolescents. The clinical exam is often benign, but intra-articular calcification can be evident on radiographs. MRI is beneficial for differentiating between potential diagnoses. Management is usually conservative, with arthroscopy reserved for symptomatic cases that fail conservative treatment. The etiology is unknown, but several theories exist. This case report describes a 16-year-old female athlete who presented with catching in her left knee and occasional pain when jumping hurdles. Radiographs were suggestive of a bony ossicle in the posterior aspect of her medial compartment. Conservative treatment provided little relief. MRI identified an intrameniscal ossicle and a posterior root tear of the medial meniscus. During arthroscopy, compression of the ossicle between the femur and tibia was visualized when the knee was positioned in terminal knee flexion and external rotation. Surgical treatment consisted of partial medial meniscectomy with excision of the ossicle and meniscal repair. The athlete gradually returned to full activity and sports. Although the exact etiology is unknown, trauma is the most likely cause. The patient’s young age and absence of calcification on prior radiographs negate degenerative and congenital causes, respectively. Meniscal ossicles in adolescents are rare but need to be considered when intra-articular calcification is present on radiographs.

## Introduction

Meniscal ossicles are rarely seen by clinicians and are usually described in case reports within the orthopedic and radiographic literature [1-24]. Prabhudesai and Richards were among the first to summarize the early case reports [1]. More recently, two relatively large case series and a systematic review have outlined typical presentations, treatments, and outcomes [2-4]. Although the incidence is unknown, a frequency of 0.15% was reported in a series of 1,287 consecutive knee MRI examinations [7]. The average age at presentation is 26 years in early literature and 51 years in later case series [1-3]. Very few reports exist in children or adolescents (birth to 17 years of age) [5-9,22]. Onset can be insidious or associated with an acute injury [1-3]. Knee pain is the most common presenting symptom, but a minority of patients complain of instability, catching, or locking [1-3]. Clinically, the exam is benign, except in the presence of concomitant pathology, which often includes a meniscal tear or anterior cruciate ligament (ACL) injury [2-4,23]. MRI is useful for differentiating between potential diagnoses that can appear as intra-articular calcification on radiographs, including an osteochondral loose body, meniscal ossicle calcification, osteochondritis dissecans, meniscal avulsion of the tibia, and chondrocalcinosis [6-11,13-14,18-20]. Management is usually conservative, with arthroscopy reserved for symptomatic cases that fail conservative treatment or that present with other pathology [1-4,15,20]. The etiology remains unknown, but three theories are postulated: congenital, degenerative, or post-traumatic causes [1-4,7,14]. The current presiding theory is a traumatic etiology consisting of either an acute injury or repetitive microtrauma [2].

We present a unique case of a medial meniscal ossicle in an adolescent patient, highly suggestive of trauma as the etiology.

## Case presentation

**Figure 1:**
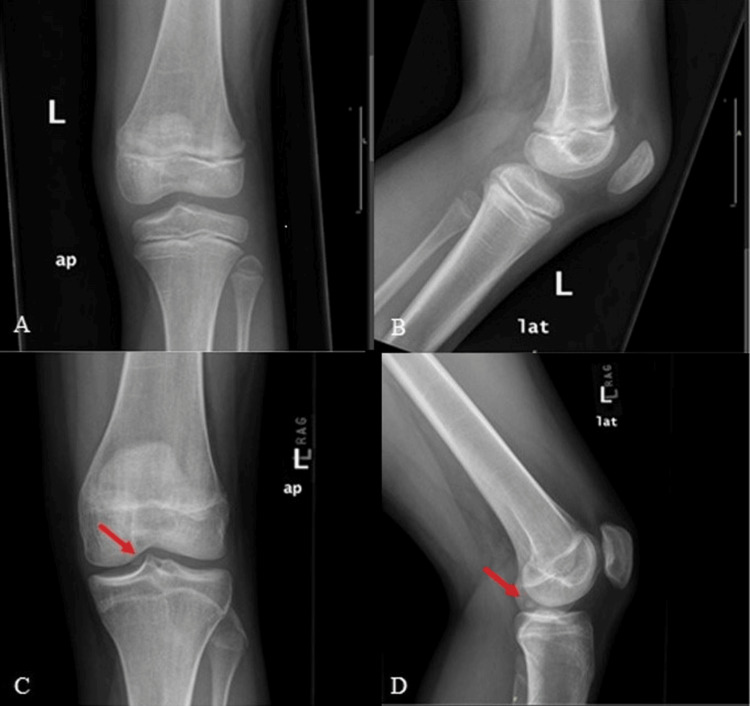
Radiographs of the left knee at nine years of age showing no evidence of a meniscal ossicle on both anterior-posterior (A) and lateral (B) views. Radiographs of the left knee at 16 years of age suggestive of a bony ossicle (arrow) in the posterior portion of the medial compartment on both anterior-posterior (C) and lateral (D) views.

**Figure 2:**
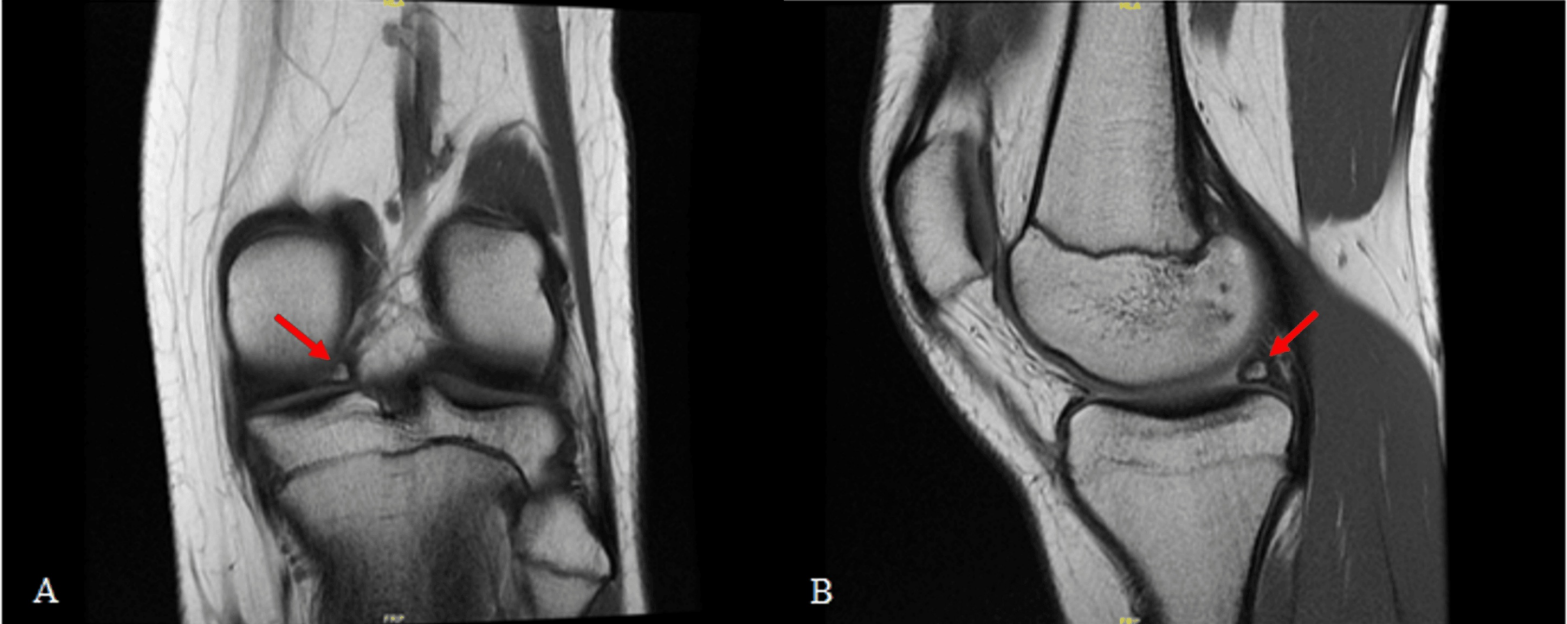
MRI of the left knee suggestive of an intra-meniscal ossicle (arrow) in the posterior horn of the medial meniscus in both coronal (A) and sagittal (B) views.

A 16-year-old female athlete presented with occasional complaints of locking and catching in her left knee for about one year after a long-jump injury that resolved without medical care. Two months prior to presentation, she began running hurdles in track and noticed an increase in pain and frequency of symptoms. These symptoms occurred when her knee was in a position of terminal knee flexion and external rotation (i.e., the position of the trail leg when jumping hurdles). She reported no swelling, stiffness, or instability. Her past medical history was unremarkable except for a discoid meniscus in her contralateral knee, which was successfully treated with saucerization and repair when she was seven years old. Clinical exam findings included full range of motion, no tenderness to palpation, no ligamentous instability, and a positive McMurray test. Radiographs of her left knee were suggestive of a bony ossicle in the posterior aspect of her medial compartment, just medial to the notch, which was not present on earlier comparative radiographs (Figure 1). MRI identified a meniscal ossicle and posterior root tear of the medial meniscus (Figure 2). The patient had tried a course of conservative treatment (rest, anti-inflammatories, stretching), but mechanical symptoms during activity persisted, so arthroscopic evaluation was suggested and pursued.

**Figure 3:**
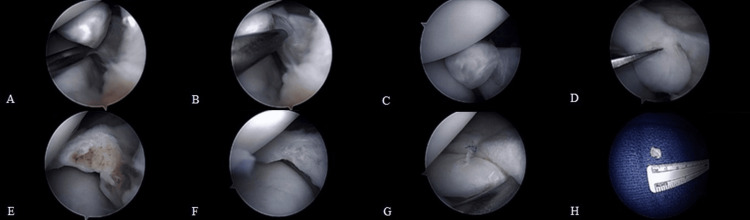
Arthroscopic images demonstrating the following: (A) a medial meniscal ossicle adjacent to the meniscal tear; (B) further demonstration of partial attachment of the ossicle to the meniscal root; (C) intra-articular ossicle compressed between the femoral condyle and tibial plateau during knee flexion; (D) excision of the meniscal ossicle; (E) cross section of the meniscal ossicle consistent with fatty marrow; (F) partial meniscectomy with removal of the ossicle; (G) meniscal repair; and (H) resected meniscal ossicle gross specimen measuring 1 cm.

At the time of arthroscopy, the posterior horn of the medial meniscus demonstrated a large, truncated amputation of the root with an intra-meniscal ossicle at its end (Figure 3). When the knee was placed in terminal flexion and external rotation, the ossicle was visualized being compressed between the medial femoral condyle and the tibial plateau, confirming the symptomatic nature of the ossicle. A partial medial meniscectomy was performed to remove the ossicle. An all-inside repair was performed to provide additional capsular attachments for the posterior horn in the hopes of preventing further tears and potential instability. The removed tissue was sent to pathology. Pathology later confirmed the removed tissue was a pedunculated portion of benign bone with a surface fibrocartilage cap and sparse fibrous tissue. The bony trabeculae were fairly unremarkable and showed interspersed adipose tissue.

After surgery, the patient was placed in a knee brace limiting knee flexion to 90 degrees and was non-weightbearing on the surgical side. After six weeks, full range of motion was allowed with advancement to full weight-bearing, including use of a stationary bike. Return to full activity and sports was permitted after three months. The patient continued to progress without complication. Approximately 18 months post-surgery, the patient was seen for right hip and left knee pain after running a 15K which was attributed to right proximal IT band syndrome and left hamstring tendinitis. She denied any locking or catching sensation in her left knee. Post-operative radiographs demonstrated no recurrence of the ossicle and no degenerative joint changes.

## Discussion

Medial meniscal ossicles in children are rare but need to be considered when intra-articular calcification is present on radiographs. In our case, abnormal radiographs led to an MRI and arthroscopy, which confirmed the presence of the ossicle and a posterior root tear of the medial meniscus. The presence of concomitant pathology, especially meniscal tears, was reported in a majority of pediatric cases (Table 1) [6-9] and in large case series and systematic reviews (Table 2) [2-4]. Although we cannot definitively say whether the ossicle or the meniscal tear caused our patient’s symptoms, we suspect the ossicle. During arthroscopy, we could see the compression and entrapment of the ossicle between the femur and tibia when the knee was placed in terminal flexion and external rotation. Furthermore, the patient only complained of pain when her knee was in this position (i.e., when she began running hurdles). Removal of the ossicle reestablished more normal joint mechanics, most likely contributing to a successful outcome.

**Table 1:** Summary of pediatric case reports in the literature. ACL: Anterior cruciate ligament; F: Female; M: Male; TAR: Thrombocytopenia - absent radius syndrome; *: Not mentioned or unknown.

Author (year)	Age years	Sex	Mechanism	Imaging at time or prior to injury	Symptoms	Other pathology
Conforty B and Lotem M (1979) [5]	16	F	Two prior surgeries	*	Pain	*
Ogden JA et al. (1994) [6]	15	M	Football	Normal	Discomfort	Meniscal fibrillar damage and posterior enlargement
	14	M	Injury	Normal	*	*
	12	M	TAR	Normal	*	Tibia vara deformity
Schnarkowski P et al.(1995) [7]	15	M	Injury	*	Pain	Posterior horn tear and ACL tear
Raustol OA et al. (2006) [8]	15	M	Soccer	*	Pain	Posterior root avulsion
Kato Y et al. (2007) [9]	16	M	Baseball	*	Pain	Posterior horn tear
Eliasberg CD et al. (2020)[22]	17	M	Trampoline	Normal	Pain	Stable small radial split in meniscus
Vangrinsven G et al. (2020) [24]	12	M	Ice-skating	Hypointense structure on MRI	Effusion	Juvenile idiopathic Arthritis

**Table 2:** Summary of patient and imaging characteristics of meniscal ossicles reported in large case series and systematic reviews in the literature. n: Sample size; ACL: Anterior cruciate ligament.

Author (year)	n	Age: Average, Range (years)	% Male	% located in medial meniscus	% with associated meniscal tear on MRI	% with abnormal ACL
Prabhudesai V, Richards PJ (2003) [1]	53	26 (12-76)	81%	92%	*	*
Bernard CD et al. (2021) [2]	45	51 (14-87)	58%	89%	93%	*
Mohankumar R et al. (2014) [3]	65	51 (23-80)	51%	89%	95%	48%
Ververidis AN et al. (2021) [4]	169	44 (12-87)	63%	*	76%	28%

The effect of the meniscal ossicle on joint mechanics may explain why some cases benefit from conservative treatment and others require surgery. Under fluoroscopy or dynamic MRI, a normal, stable ossicle can be seen moving with the tibial plateau during knee flexion and extension [9,19-21]. This was the case with a 20-year-old soccer player who achieved good results with conservative treatment alone [20]. In our case, the ossicle altered normal joint mechanics as seen during arthroscopy, so positive results were achieved only with its removal. We therefore concur with Liu SH et al., that it is important to investigate the effect of the meniscal ossicle on joint kinematics whether through fluoroscopy, dynamic MRI, or arthroscopy to help guide treatment [20].

Compared to the literature, this case had some unique aspects. Most of the eight cases previously reported in children involved male athletes, with known injury, concomitant pathology, and whose primary complaint was knee pain (Table 1) [5-9,22]. Our case differed in gender, onset, and chief complaint. When compared to the large case series and systematic reviews of meniscal ossicles, our case differed in terms of age at presentation (16 years vs. 51 years) (Table 2) [1-4]. The absence of the meniscal ossicle from prior imaging is somewhat unique, representing only 32% (7/22) of patients in a prior study [1]. However, this was also reported in pediatric cases described by Ogden JA et al. and Eliasberg CD et al., and in several adult cases [1-2,6,19,22].

The etiology of meniscal ossicle is under debate and includes congenital, degenerative, or post-traumatic causes [1-4,7,14]. Eliasberg CD et al. also described a meniscal ossicle developing after a meniscal root repair augmented with bone marrow aspirate concentrate [22]. In our case, the ossicle as a congenital vestigial structure is unlikely because it was absent from prior imaging. Our patient is also too young for degenerative changes, which typically occur in the third decade of life or later [3]. Trauma appears to be the likely cause, whether it be from her long-jump injury, a meniscal tear, or repetitive trauma sustained when running hurdles. In retrospect, the patient may have sustained a meniscal root tear with subsequent ossicle formation from the initial long-jump or other associated trauma. However, repetitive trauma is also plausible. The patient did not present with many of the signs or symptoms commonly associated with a posterior root tear, such as abrupt onset of more severe pain, a discrete event preceding the pain, tenderness at the joint line, effusion, and diminished range of motion.

The strengths of this case report include a relatively unique presentation of a rare condition, visualization of altered joint mechanics during arthroscopy, and trauma as the most likely etiology. Limitations include those common with any retrospective review, namely that data is restricted to what is recorded in the chart.

## Conclusions

Meniscal ossicles are very rare in children and adolescents but should be considered when intra-articular calcification is present on radiographs. MRI can identify the ossicle, typically located in the posterior horn of the medial meniscus. It is also important to investigate the effect of the meniscal ossicle on joint kinematics. When conservative treatment fails, surgical removal of the ossicle should be considered, especially if the effect of the ossicle on joint kinematics provides a plausible explanation for the symptoms.
